# How community ecology can improve our understanding of cholera dynamics

**DOI:** 10.3389/fmicb.2014.00137

**Published:** 2014-04-02

**Authors:** Guillaume Constantin de Magny, Nur A. Hasan, Benjamin Roche

**Affiliations:** ^1^Maladies Infectieuses et Vecteurs Ecologie, Génétique, Evolution et Contrôle, Institut de Recherche pour le Développement, UMR 224 IRD-5290 CNRS-UM1-UM2Montpellier, France; ^2^Maryland Pathogen Research Institute, University of Maryland, College ParkMD, USA; ^3^Unité de Modélisation Mathématique et Informatique des Systèmes Complexes, Institut de Recherche pour le Developpement, UMI IRD/UPMC 209Bondy, France

**Keywords:** cholera, ecology, metagenomics, genetics, pathogen

## Abstract

Understanding the seasonal emergence and reemergence of cholera is challenging due to the complex dynamics of different protagonists. The abundance of *Vibrio cholerae*, the causative agent of cholera and a natural inhabitant of aquatic environments, fluctuates according to abiotic, and biotic factors. Among the biotic factors, the zooplankton community dynamics has been suggested to play a pivotal role in the survival, persistence, and natural competence of *V. cholerae*. However, factors regulating *V. cholerae* population structure and seasonal dynamics are still not fully understood. Investigation of the temporal shifts and variability in aquatic community composition in relation to the occurrence or abundance of *V. cholerae* appears very promising yet remained underexplored. Recent advances in metagenomics, facilitated by high-throughput ultra deep sequencing, have greatly improved our ability for a broader and deeper exploration of microbial communities including an understanding of community structure, function, as well as inter- and intra-specific competitions. Here, we discuss possible areas of research focusing how combination of community ecology and metagenomic approaches could be applied to study the cholera system.

The diarrheal disease, cholera, is caused by the pathogenic bacteria *Vibrio cholerae*; affecting hundreds of thousands of people every year with thousands of deaths (World Health Organization, 2010). Depending on the health status, infection occurs due to the ingestion of 10^3^–10^9^ cells of pathogenic *V. cholerae* (either O1 or O139) through contaminated food and water. After the ingestion, the microbe has to pass through acidic conditions of the human stomach and the immune system defenses to reach the small intestine, where it attaches, and begins to produce cholera toxin (CT). Sufficient quantities of CT cause severe diarrhea and shedding of the pathogen, up to 10^7^ per infected individual per stool (Zahid et al., 2008), into the environment facilitating spread of the disease.

Outside the human host, *V. cholerae* is an autochthonous member of natural aquatic environments such as lakes, rivers, estuaries, and the ocean, which serve as the principal natural reservoir for this organism. Ecologically, vibrios play an important role in the degradation of organic matter and act as a link that transfers dissolved organic carbon to higher trophic levels of the marine food web (Grossart et al., 2005).* V. cholerae* is a facultative anaerobic, asporogenous, gram-negative rod with capability of respiratory and fermentative metabolism. It is oxidase positive, reduces nitrate, and motile by a single polar, sheathed flagellum (Kaper et al., 1995). The pathogenic and non-pathogenic strains of *V. cholerae* are globally distributed in aquatic environments (Lipp et al., 2002). Historically, cholera is endemic in the Bengal basin with outbreaks occurring elsewhere due to lack of access to clean water and sanitation facilities (World Health Organization, 2010). The role of climate and other abiotic factors on cholera has been well investigated (Lipp et al., 2002) but approaches taken to date to study *V. cholerae* have fallen short in separating the role of abiotic and biotic factors. Therefore, we are advocating the need for both genomics and community ecology approaches to elucidate such relationships.

How *V. cholerae* survives in two very distinct habitats, human hosts, and aquatic ecosystems, and mechanisms by which it periodically emerges as a human pathogen are compelling questions in the ecology of this species. Understanding the ecology of *V. cholerae* is, however, challenging because of the complex dynamics of different protagonists. In this paper, we will explore how knowledge from multiple different disciplines like ecology, genomics, and modeling, might contribute to establish an ecological framework of *V. cholerae* to provide critical insights in our understanding of this bacterium.

A widely accepted concept in theoretical ecology of *V. cholerae* is *the niche theory*. The niche theory is defined by three main factors that contribute to population growth rate: resources, the physical environment, and natural enemies (Vandermeer, 1972). How a species respond to these factors, spatially, and temporarily, determines its ability to invade, persist, and occasionally become dominant. This appears as a critical phenomenon in understanding the ecology of *V. cholerae*.

The first factor is resources. For *V. cholerae*, this factor is strongly linked to the availability of chitin in the natural environment. In the aquatic ecosystem, chitin is the most abundant polysaccharide and the principal component of zooplankton exoskeleton. *V. cholerae* is strongly associated with plankton, forming commensal relationships with chitinous organisms that are dominant among zooplankton populations (i.e., copepods, amphipods, and other crustaceans; Colwell and Huq, 1994). The copepod exoskeleton has been shown to support large population of vibrios, including the pathogenic *V. cholerae* (Colwell et al., 1981; Huq et al., 1983; Tamplin et al., 1990). An advantage of epibiotic organisms, such as *V. cholerae*, on biotic substrates, is their proximity to available nutrients. *V. cholerae* cells living on highly mobile zooplanktons are less likely to be nutrient limited than “free-living” or planktonic cells in the environment.

The commensal relationship between vibrios and chitinous organisms, like copepods, has important consequences (Pruzzo et al., 2008). This relationship represents a useful model to investigate the role of primary habitat selection in developing pathogenicity traits of the bacteria primarily inhabiting the aquatic environment. It has also been reported that *V. cholerae* becomes naturally competent and amenable to serotype conversion on chitin surfaces (Meibom et al., 2005). Hence, the ability of chitin to support both genetic evolutionary diversity and growth of cholera organisms in the environment strongly implicates copepods as a critical component in the environmental lifestyle of this human pathogen. Indeed, zooplanktons comprise a broad assortment of ecologically important heterotrophic groups. The composition of zooplankton community changes constantly during an annual cycle, with dramatic differences in species composition of freshwater and marine zooplankton communities. Thus, the plankton species composition plays a pivotal role in *Vibrio* seasonality, as seen, i.e., in waters off the Georgia (U.S.) coast, highlighting the complex relationship between seasonal shifts in plankton composition and number of vibrios in the aquatic environment (Turner et al., 2009).

Statistical model parameter estimates have indicated a strong direct relationship between *Vibrio* concentration and the relative abundance of copepods in the >200 μm fraction. Recently, the incidence of cholera and the occurrence of pathogenic *V. cholerae* with diverse zooplankton taxa were studied in rural areas of Bangladesh (Constantin de Magny et al., 2011). Chitinous zooplankton communities of several water bodies were analyzed in order to understand the interaction of the zooplankton population composition with the population dynamics of pathogenic *V. cholerae* and the incidence of cholera. Two dominant zooplankton groups, namely, rotifers and cladocerans, were consistently associated with detection of *V. cholerae* and/or occurrence of cholera cases. Specifically, the presence of rotifer, *Brachionus angularis,* was significantly associated with the presence of pathogenic *V. cholerae.* Local differences indicated some subtle ecological factors influencing the interactions between *V. cholerae*, its plankton hosts, and incidence of cholera. Like the determination of a potential keystone species (Mills and Doak, 1993), or assemblage of species associated with the occurrence of *V. cholerae*, understanding the community dynamics (diversity and abundance) of zooplanktons in the natural aquatic environment could be a key element in deciphering the complete picture of *V. cholerae* ecology.

The second factor of the niche theory for *V. cholerae* is the physical environment. For most *Vibrio* sp., temperature and salinity are two major abiotic factors strongly associated with their abundance (Lipp et al., 2002). Materna et al. (2012) remarkably investigated the shape and evolution of the fundamental niche of marine *Vibrio* by combining theoretical and experimental approaches on 2-dimensional niche shape associated with temperature and salinity, the two major factors in *Vibrio* ecology. It was found that for the studied marine *Vibrio* strains, ecological range, in reality, occupies a limited section of their potential niche (Materna et al., 2012). In addition to the physical drivers of the population growth, this bacterial species, like other gram-negative bacteria, has a selective advantage in its ability to enter a dormant stage when environmental conditions are unfavorable for active growth and cell division. This state is termed the viable but non-culturable (VBNC) state (Cottingham et al., 2003; Takeda, 2011). This represents another important aspect of the ecological niche of *V. cholerae,* namely its adaptability to fluctuations of the abiotic conditions.

The VBNC state of bacteria is defined as the state where cells remain viable but cease to grow or divide, on, or in, routinely used bacteriological media. Even if it is still in debate, evidences of the reversibility of this state back to active growth and cell division have accumulated (Senoh et al., 2010; Takeda, 2011). This non-culturability associated with the ability to aggregate on biofilm, even after having been non-culturable for more than one year (Alam et al., 2007), is another important aspect in the ecology of *V. cholerae.* Several studies have already demonstrated the importance of the VBNC state in the seasonal epidemics of cholera (Colwell et al., 1985, 1996; Alam et al., 2006a,b; Alam et al., 2007; Asakura et al., 2007). A recent study (Mishra et al., 2012), however, demonstrated a vital role of the VBNC state in cholera epidemiology manifesting an active expression of traits necessary for both viability, stress response, virulence, and colonization of the bacterium.

Yet another factor of the ecological niche of *V. cholerae* is the biotic antagonists. Among them, the lysogenic filamentous phage, CTXΦ, is found in toxigenic *V. cholerae* (Waldor and Mekalanos, 1996) and encodes the CT. The *V. cholerae* pathogenicity island, VPI, another antagonist, encodes a toxin co-regulated pilus that functions as a colonization factor, and a receptor for CTXΦ. It has been hypothesized that lytic bacterial viruses are responsible for the decline in the incidence of cholera during seasonal outbreaks (Faruque et al., 2005b), limiting *V. cholerae* population densities through predation. It has been suggested that the increased environmental phage abundance is the result of a host-mediated phage amplification during the cholera epidemic, decreasing the load of environmental *V. cholerae* resulting in the collapse of the epidemic (Faruque et al., 2005a). This predator/prey relationship have been debated due to the lack of evidences distinguishing the effect of growth-limiting resources and the lytic activity of the phage restricting the densities of *V. cholerae* population (Wei et al., 2010). The alternative hypothesis states that phage-resistant *V. cholerae* mutants are less fit than wild-type, possessing a number of characteristics that are likely to further reduce capacity to be maintained in natural populations and may be avirulent in human hosts (Wei et al., 2010). The role of the combined effect of growth resources and the lytic activity of phages on *V. cholerae* population abundances need to be clarified for better understanding of bacterial population dynamics driver.

In addition, it appears that *V. cholerae* does not give up without a fight. *V. cholerae*, like other Gram-negative bacteria, utilizes accessory virulence factors, e.g., the type VI secretion system (T6SS), to provide advantages in intraspecific and interspecific competition (Unterweger et al., 2012). It is hypothesized that the T6SS and toxin translocation play critical roles for *V. cholerae* to outcompete other bacteria and phagocytic cells. Thus, it persists in human hosts and in the environment (Miyata et al., 2010). Furthermore, it was shown that in order to successfully colonize organic matter in the marine environment, *V. cholerae* must compete against a high concentration of diverse bacteria that reside on the surface of particles. The interspecies antagonistic interactions involving allelochemicals can influence *V. cholerae* particle colonizations. These interactions can also be temperature sensitive (Long et al., 2005). However, the definite role of the microbial (viral and bacterial) community on *V. cholerae* abundance is still unknown. As microbes are interacting with each other, at an individual scale (Hajishengallis et al., 2012), or at a population level (Telfer et al., 2010), overlooking such interaction may lead to a partial understanding of the contributing factors. However, microbial communities are typically very diverse and complex, therefore, characterization of species diversity and the complex network of species interactions within these communities also pose a technical challenge to the scientific community.

The advent of next-generation sequencing technologies and bioinformatics opened up the door to a wide new era of research exploration and has offered an unprecedented opportunity to fill an existing knowledge gap in community ecology. Recent advances of such technologies and tools have already revolutionized the field of metagenomics, allowing a much more accurate, and detailed description of microbial communities. As these technologies and tools are becoming widely available and accessible, their application is becoming the cornerstone in addressing the outstanding hypotheses in various domains like biogeography as well as furthering our understanding of how ecological communities assemble, evolve, and function. Next-generation high-throughput sequencing (HTS) methods have already been proven as an outstanding way to describe microbial biodiversity and community ecology in a variety of systems and environments. By definition, an individual ecosystem is composed of populations of organisms, interacting within communities, and contributing to the cycling of nutrients, and the flow of energy. Conventional methods of sampling ecosystems are unquestionably biased toward the highly abundant and easy to characterize members of the community where HTS methods facilitate the opportunity to study every member of the community either individually, or as a whole, to reflect the entire microbial system. Additionally, decomposition and nutrient cycling are key processes in ecosystem functioning. Both are fundamental to ecosystem biomass production, and mostly operated by microbes, more precisely bacteria. Understanding microbial community dynamics in ecosystems is, therefore, one of the key elements deciphering ecosystem functioning. As *V. cholerae* survives in two very distinct habitats, human hosts, and aquatic ecosystems, it is, therefore, critical to investigate the influence of microbial population dynamics on seasonal emergence and reemergence of *V. cholerae*, especially to understand what happens in the community when there is an outbreak.

Historically, the first metagenomics research was focused on cultured soil microorganisms (Handelsman et al., 1998). Rapidly, it became a very promising source of knowledge in understanding the dynamics of microbial population, the interaction between pathogens, and the microbial community, and how they evolve in different habitats (e.g., host, vector, reservoir). Recently, Monira et al. (2013) explored gut microbiota of children suffering from cholera and during disease recovery. From the nine children included in the study, they reported *V. cholerae* accounted for 35% (minimum 5%, maximum 63%) of the total gut microbiota at day 0, with *V. cholerae* was the predominant species in six of the nine children (Monira et al., 2013). During the recovery, the effect of antibiotic therapy demonstrated a rapid shift in bacterial diversity affecting the top ten bacterial families, which accounted for more than 85% of the bacterial flora during cholera. Furthermore, cholera infection appears to induce expulsion of major commensal bacteria belonging to the phyla, e.g., Bacteroidetes, Firmicutes, and Actinobacteria, facilitating the colonization of the gut by harmful bacteria belonging to the phylum Proteobacteria (Monira et al., 2013).

Does this phenomenon of interspecific competition observed in the human gut during cholera infection also occur in the second distinct habitat, the aquatic ecosystem? Other compelling questions in microbial community ecology also arise from this. What is the structure and composition of the microbial communities in the aquatic environment directly sourced by the human populations for their daily needs? How did these microbial communities evolve over time? What space does *V. cholerae* occupy in the communities and how does it impact the cholera dynamics (emergence, transmission)? How similar microbial communities are in the aquatic environments where cholera is endemic, epidemic, or benign? Ongoing research in Bangladesh based on the limited scale application of 16S rRNA gene survey suggests that the abundance and diversity of the microbial community including *Vibrio* populations exhibited a complex seasonal distribution driven by temporal fluctuations of environmental parameters, such as sea surface temperature, and indicates that a specific consortium among the indigenous population, and their interaction might be crucial in responding to changes in environmental parameters that allow *V. cholerae* to overcome the natural competition and emerge in epidemic proportions.

Translating these capacities of a metagenomics approach to the study of *V. cholerae*, we suggest that such approach could provide crucial insights to infer competition processes within microbial communities. While studies thus far have focused mostly on the environmental dimension of *V. cholerae* niche (Preheim et al., 2011a,b; Materna et al., 2012), adopting metagenomics based community ecology approach could add a critical, perhaps keystone, dimension to the study of *V. cholerae*, and the robustness of predictive models could improve substantially by incorporating such a fundamental dimension.

Potentially, the integration of all the knowledge about the disease cholera, and the ecology of *V. cholerae*, should lead to an End-To-End model of cholera. End-to-End models are integrated models of an ecosystem, which encompasses climate, various ecosystem components, and in some cases human interaction with the ecosystem. These models are developed to describe, understand, and predict ecosystem dynamics. To this extent, integrating an ecosystem component in such a model, and the microbial community context involving natural enemies of bacteria that influence strongly the kinetics of *V. cholerae* in the environment and, consequently, in human populations, would be crucial in achieving the objective of such a predictive tool. This is in contrast to the traditional approach or the old paradigm of climate prediction for human health that seeks correlations between climatic variables, morbidity, mortality, exacerbation of chronic conditions, population-level outbreaks, or other indicators that are precursors to an outbreak. Additionally, there is a critical need to plug ecological niche modeling (ENM) into this approach. ENM is often used to inform conservation efforts (Peterson and Robins, 2003), to describe species potential range (Meentemeyer et al., 2004), and is increasingly being used to identify species of public health concern (Mullins et al., 2011). Advances so far in environmental predictions for human health can be illustrated by the excellent analogy for Earth System interactions provided by El Niño. Examples range from heat and cold wave related mortalities and morbidities to cholera, malaria, dengue, and meningitis. The impacts of global changes are already manifesting themselves in global indicators such as temperature and sea level rise, but the impacts on populations occur through local changes in weather, ecology, water resources, barometric pressure, etc.

The salience, reliability, and legitimacy of a successful prediction system will depend on filling the gaps in mechanistic linkages from changes in climate to effects on human health and well-being. Thus, simple empirical approaches of the past must be steered toward more mechanistic methodologies to identify the drivers from all fronts, e.g., environment, agriculture, and water. These include genetic, chemical, and biological factors, such as microbial contamination pathways, human behavior, exposure, social, and health systems infrastructure, demographics, and other measures of vulnerability that affect the onset, spread, and exacerbation of disease, and the resources needed (water, food, shelter) to maintain health. Networked phenomena including the flow and potency of pathogens will be a useful component. In addition to geographic measures, vulnerability, and risk clearly depend on various socio-economic and demographic factors. Regional Earth System Prediction can account for these factors in an adaptive, learning-by-doing mode of model-data synthesis. More importantly, these disparate pieces have to be integrated into Earth System models, especially in the high resolution RESP framework (Murtugudde, 2009) with goal-oriented observational monitoring networks.

## Conflict of Interest Statement

The authors declare that the research was conducted in the absence of any commercial or financial relationships that could be construed as a potential conflict of interest.
